# Costs and benefits of rapid screening of methicillin-resistant *Staphylococcus aureus *carriage in intensive care units: a prospective multicenter study

**DOI:** 10.1186/cc11184

**Published:** 2012-02-07

**Authors:** Marjan Wassenberg, Jan Kluytmans, Stephanie Erdkamp, Ron Bosboom, Anton Buiting, Erika van Elzakker, Willem Melchers, Steven Thijsen, Annet Troelstra, Christina Vandenbroucke-Grauls, Caroline Visser, Andreas Voss, Petra Wolffs, Mireille Wulf, Ton van Zwet, Ardine de Wit, Marc Bonten

**Affiliations:** 1 Department of Medical Microbiology, University Medical Center, Heidelberglaan 100, Utrecht, 3584 CX, The Netherlands; 2 Department of Internal Medicine and Infectious Diseases, University Medical Center, Heidelberglaan 100, Utrecht, 3584 CX, The Netherlands; 3 Laboratory for Microbiology and Infection Control, Amphia Hospital, Molengracht 21, Breda, 4818 CK, The Netherlands; 4 Department of Medical Microbiology and Infection Control, VU University Medical Center, De Boelelaan 1118, Amsterdam, 1081 HZ, The Netherlands; 5 Department of Medical Microbiology, Hygiene and Infection Prevention, Slingeland Hospital, Kruisbergseweg 25, Doetinchem, 7000 AD, The Netherlands; 6 Public Health Laboratory Tilburg, St. Elisabeth Hospital, Hilvarenbeekseweg 60, Tilburg, 5022 GC, The Netherlands; 7 Department of Medical Microbiology and Infection Prevention, Haga Hospital, Leyweg 275, The Hague, 2545 CH, The Netherlands; 8 Department of Medical Microbiology, Radboud University Nijmegen Medical Center, Geert Grooteplein-Zuid 10, Nijmegen, 6525 GA, The Netherlands; 9 Laboratory of Medical Microbiology and Immunology, Diakonessenhuis, Bosboomstraat 1, Utrecht, 3582 KE, The Netherlands; 10 Department of Medical Microbiology, Academic Medical Center, Meibergdreef 9, Amsterdam, 1105 AZ, The Netherlands; 11 Department of Medical Microbiology and Infectious Diseases, Canisius Wilhelmina Hospital, Weg door Jonkerbos 100, Nijmegen, 6532 SZ, The Netherlands; 12 Department of Medical Microbiology, CAPHRI, Maastricht University, P. Debyelaan 25, Maastricht, 6229 HX, The Netherlands; 13 Laboratory for Pathology and Medical Microbiology, PAMM Institute, De Run 6250, Veldhoven, 5504 DL, The Netherlands; 14 Laboratory for Medical Microbiology and Immunology, Alysis Zorggroep, Wagnerlaan 55, Arnhem, 6815 AD, The Netherlands; 15 National Institute of Public Health and the Environment, Antonie van Leeuwenhoeklaan 9, Bilthoven, 3721 MA, The Netherlands; 16 Julius Center for Health Sciences and Primary Care, University Medical Center,Heidelberglaan 100, Utrecht, 3584 CX, The Netherlands

## Abstract

**Introduction:**

Pre-emptive isolation of suspected methicillin-resistant *Staphylococcus aureus *(MRSA) carriers is a cornerstone of successful MRSA control policies. Implementation of such strategies is hampered when using conventional cultures with diagnostic delays of three to five days, as many non-carriers remain unnecessarily isolated. Rapid diagnostic testing (RDT) reduces the amount of unnecessary isolation days, but costs and benefits have not been accurately determined in intensive care units (ICUs).

**Methods:**

Embedded in a multi-center hospital-wide study in 12 Dutch hospitals we quantified cost per isolation day avoided using RDT for MRSA, added to conventional cultures, in ICUs. BD GeneOhm™ MRSA PCR (IDI) and Xpert MRSA (GeneXpert) were subsequently used during 17 and 14 months, and their test characteristics were calculated with conventional culture results as reference. We calculated the number of pre-emptive isolation days avoided and incremental costs of adding RDT.

**Results:**

A total of 163 patients at risk for MRSA carriage were screened and MRSA prevalence was 3.1% (*n *= 5). Duration of isolation was 27.6 and 21.4 hours with IDI and GeneXpert, respectively, and would have been 96.0 hours when based on conventional cultures. The negative predictive value was 100% for both tests. Numbers of isolation days were reduced by 44.3% with PCR-based screening at the additional costs of €327.84 (IDI) and €252.14 (GeneXpert) per patient screened. Costs per isolation day avoided were €136.04 (IDI) and €121.76 (GeneXpert).

**Conclusions:**

In a low endemic setting for MRSA, RDT safely reduced the number of unnecessary isolation days on ICUs by 44%, at the costs of €121.76 to €136.04 per isolation day avoided.

## Introduction

Nosocomial infections caused by methicillin-resistant *Staphylococcus aureus *(MRSA) have been associated with increased mortality and high health care costs [1,2]. There is considerable geographic variation in the prevalence of nosocomial MRSA infections. In intensive care units (ICUs) in the US the prevalence of MRSA among clinical *S. aureus *isolates is over 55% [3,4], while in countries with a national search and destroy policy for MRSA, such as Scandinavian countries and the Netherlands, the prevalence among bacteremia isolates is still around 1% [5]. Pre-emptive isolation of patients considered at high risk for MRSA carriage is considered a cornerstone of such a control policy and has been shown to reduce ICU acquired MRSA infections in medical ICUs [6]. However, the vast majority of patients considered at increased risk for carriage will not be colonized with MRSA, yielding considerable amounts of unnecessary isolation days as conventional microbiological culture methods have a diagnostic delay of three to five days. Isolation measures are costly [7,8] and may compromise the quality of patient care [9].

Rapid molecular screening for MRSA carriage may reduce the logistical and financial burdens associated with pre-emptive isolation of ICU patients. However, the costs and effects of such diagnostic tests have not been determined for use in ICUs [10]. Therefore, we quantified costs and benefits of two rapid screening tests for MRSA on ICUs in a multi-center study in the Netherlands.

## Materials and methods

### Study design and setting

A prospective multi-center study was performed in 12 Dutch hospitals (5 university hospitals, 7 teaching hospitals) between December 2005 and June 2008. The effects of rapid diagnostic testing (RDT) of MRSA, using PCR added to screening with conventional microbiological culture methods for patients eligible for MRSA screening, were evaluated. Two real time PCR assays were subsequently evaluated: BD GeneOhm™ MRSA PCR (previously known as IDI-MRSA) ('IDI', BD Diagnostics, San Diego, CA USA) between December 2005 and May 2007, and Xpert MRSA assay ('GeneXpert', Cepheid, Sunnyvale, CA USA) between April 2007 and June 2008. PCR-based testing was compared to concurrently performed conventional microbiological techniques. We will refer to the study period using BD GeneOhm™ MRSA PCR as the 'IDI study' and to the study period using Xpert MRSA assay as the 'GeneXpert study'.

Eligibility for screening was based on the risk profile for MRSA carriage, as defined in Dutch guidelines (Table 1). Proven MRSA carriers were not included. Before onset of the study, patients categorized as at high risk for MRSA carriage were screened and pre-emptively isolated until conventional microbiological culture results had demonstrated absence of MRSA. During isolation, patients were nursed in a single-patient room (preferably with anteroom) and with barrier precautions. This practice has been used for more than 20 years and is routine in all Dutch hospitals. The current intervention implied that continuation (or discontinuation) of pre-emptive isolation was decided immediately upon the results of PCR testing, which was performed as soon as possible in patients meeting the screening criteria. The IDI and GeneXpert study included patients in both the ICU and nursing departments. However, because of lack of experience with RDT for MRSA carriage, the results of RDT were initially not used to discontinue isolation measures in ICU patients. Yet, from October 2006 on, isolation measures were also discontinued upon RDT results in ICUs. Results of the study in nursing departments have been published elsewhere [10].

**Table 1:** Patients considered at high risk for MRSA colonization and eligible for screening and pre-emptive isolation according to the guideline of the Dutch Working Party on Infection Prevention^a^

Patients transferred from a foreign hospital who	1) have been admitted there for more than 24 hours, or
	2) have undergone surgery, or
	3) had a catheter, or
	4) were intubated, or
	5) have wounds or infections such as abscesses or furunculosis
Patients transferred from a Dutch hospital or nursing home with an uncontrolled MRSA outbreak	
Patients who are contacts of an unexpected MRSA carrier	
Patients treated for MRSA carriage when control cultures are not performed yet or unknown	
Children who had been adopted from foreign countries	
Patients who have professional exposure to living pigs on pig farms or patients living on a pig farm^b^	

^a^MRSA guideline: http://www.wip.nl/free_content/richtlijnen/mrsa%20ziekenhuis080310.pdf. ^b^During the study (July 2006) added as a risk factor. MRSA, methicillin-resistant *Staphylococcus aureus*.

The institutional review board was informed although approval for the study and informed consent were not required as the intervention, screening for MRSA, concerns usual care, provides direct benefit to patients and is part of the regular infection control program conducted by the department of hospital hygiene and infection control.

### Cost analysis

The primary endpoint was the cost per isolation day avoided with rapid MRSA screening tests when added to conventional screening. Computerized reporting of all steps in the microbiology laboratory and recording the time point of start and discontinuation of isolation by the infection control practitioners allowed exact determination of turn around times (TATs). Isolation days avoided are the number of additional isolation days should PCR not have been performed, and isolation would only have been discontinued on negative conventional cultures results. It was determined using the time of availability of conventional culture results. If the PCR result was positive, isolation measures were continued awaiting conventional cultures, and when cultures appeared negative, isolation measures were discontinued with no isolation days saved. Isolation days avoided by patients in the IDI study for whom PCR testing was not used to change isolation measures, were calculated upon the hypothetical scenario that these results had been used in decision making on termination of isolation. When the exact moment of the start or discontinuation of isolation was missing the median of the duration of isolation using the IDI, GeneXpert or conventional culture was imputed. We determined incremental costs of adding rapid screening tests to the current Dutch MRSA policy. Incremental costs of rapid screening tests were calculated and included costs attributed to rapid screening and costs because of false negative test results. The incremental costs were divided by the number of avoided isolation days to calculate the cost per isolation day avoided. Costs were calculated from the hospital perspective. A detailed description of the cost analyses has been published elsewhere [10].

### Microbiological analyses

#### Samples

After meeting the eligibility criteria, swabs from the anterior nares, throat, perineum and, if present, wounds, catheter insertion sites, sputum and urine samples (in case of an indwelling urinary catheter) were obtained as soon as possible. Swabs in liquid Stuart transport media (Becton Dickinson) were used for MRSA PCR and these samples were taken firstas it was important to avoid, as much as possible, a false negative PCR. *Subsequently *swabs (according to local protocol, mainly cotton swabs) were taken for conventional culture. Specimens were transported at room temperature and refrigerated until being processed.

#### Conventional microbiological MRSA screening

Specimens for conventional microbiological cultures were processed according to the guidelines of the Dutch Society of Medical Microbiology [11], which includes a broth enrichment step for all swabs, combined with selective and non-selective agar plates.

#### MRSA PCR

During the IDI study specimens were processed with the BD GeneOhm™ MRSA PCR assay run on the SmartCycler platform (Cepheid, Sunnyvale, CA USA). Specimen preparation adaptations were made for swabs from wounds (50 μl cell suspension added to lysis tube, lysis tube not centrifuged) and urine and pus (first centrifuged for 10 minutes and subsequently sample buffer was added to the pellet). The PCR assay was performed according to manufacturers' manual, with an additional freeze-thaw cycle of the prepared lysates to reduce inhibitory effects of interfering substances. The laboratory could decide on the procedure as long as optical control was performed to confirm freezing. In the GeneXpert study the Xpert MRSA assay run on a 4-site GeneXpert^® ^system (Cepheid, Sunnyvale, CA USA) was used in accordance with manufacturers' protocol. Nose, throat and perineum specimen were processed separately, and additional specimens, when present, were pooled in the fourth cartridge.

On a patient level, PCR-based screening results were considered positive if at least one PCR result was positive and were considered negative if the nasal swab and at least one other test result were negative (and the other sites negative or non-conclusive). In case of a non-conclusive PCR result of the nasal swab, the overall test result for that patient was considered non-conclusive and isolation was continued until conventional cultures were negative or until a second PCR test performed on a new nasal swab was negative. Results, both positive and negative, were immediately reported to the wards. MRSA PCR was performed within 24 hours on working days, and not in weekends and on holidays (except for five hospitals during the GeneXpert study). Final results of conventional cultures were considered as the gold standard. Therefore, isolation measures based upon a positive PCR result were withdrawn when conventional culture results were negative.

### Statistical analysis

Test characteristics were determined at the patient level based on a combination of the results from all anatomical sites sampled. For determination of test characteristics patients were either MRSA negative (including those with non-conclusive results) or positive.

Continuous variables were compared using the Mann-Whitney *U *test; categorical variables were compared with the χ^2 ^test and Fisher exact test. All analyses were performed using SPSS.

## Results

### Patient population and test characteristics

**Figure 1:**
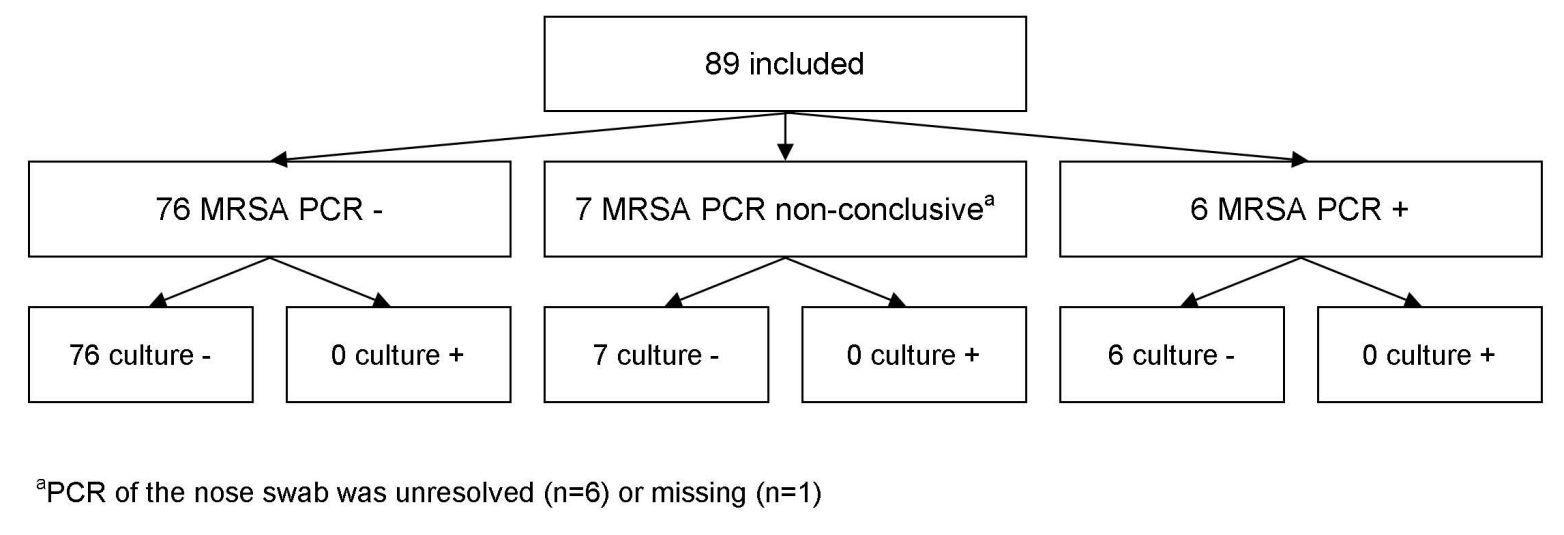
**Flowchart of patients included in the IDI study**. ^a^Contact screening patients were only assessed when the contact screening was of limited size as the number of available slots on the SmartCycler is 14 (maximum of four patients). ^b^PCR of the nose swab was unresolved. PCR, polymerase chain reaction.

**Figure 2:**
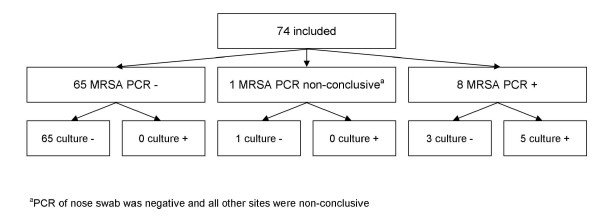
**Flowchart of patients included in the GeneXpert study**. ^a^PCR of the nose swab was unresolved (*n *= 12) or the nose swab was negative and all other sites were non-conclusive (*n *= 2). PCR, polymerase chain reaction.

A total of 163 patients were included yielding 941 screening samples (163 nares, 163 throat, 163 perineum, 129 wound, 85 urine, 52 sputum, 186 catheter insertion sites, drains and other samples): 5.8 sites were tested per patient. Eighty-nine patients were screened with BD GeneOhm™ MRSA PCR (Figure 1) and 74 with Xpert MRSA assay (Figure 2). In total 787 MRSA PCRs were performed, 519 (5.8 per patient, all specimens processed separately) and 268 (3.6 per patient, including pooled specimens) in the IDI and GeneXpert studies, respectively. Numbers of patients included per hospital ranged from 1 to 57. Contact with an MRSA carrier was the most important reason for screening (Table 2). The prevalence of MRSA carriage, based upon conventional microbiological cultures, was 3.1% (*n *= 5 patients), with the highest carriage rate among those screened because of contact with pigs. Using the results of conventional cultures as reference, sensitivity, specificity, and positive (PPV) and negative predictive (NPV) values for detecting MRSA (at the patient level) were 100% (95% confidence interval (CI) 0.46 to 1), 94.3% (95% CI 0.89 to 0.97), 35.7% (95% CI 0.14 to 0.64) and 100% (95% CI 0.97 to 1) for both screening tests together. For IDI the specificity and NPV were 93.3% (95% CI 0.84 to 0.97) and 100% (95% CI 0.94- to 1), respectively. As there were no patients with MRSA detected in the IDI study, sensitivity and PPV could not be determined. For GeneXpert sensitivity, specificity, PPV and NPV were 100% (95% CI 0.46 to 1), 95.7% (95% CI 0.87 to 0.99), 62.5% (95% CI 0.26 to 0.90) and 100% (95% CI 0.93 to 1), respectively.

**Table 2:** Demographic and clinical characteristics of patients

Characteristics	(Number = 163)
Men, Number (%)	97 (59)
Age, median (IQR), years	53 (20.4-67.9)
Hospital discharge alive, Number (%)	134 (82)
Reason for MRSA suspicion, Number (%)	
Contact with MRSA carrier	91 (56)
Treatment in foreign hospital	50 (31)
Contact with pigs	11 (7)
Other	11 (7)
MRSA carriage, Number (%)	
Overall	5/163 (3)
Contact with MRSA carrier	1/91 (1)
Treatment in foreign hospital	1/50 (2)
Contact with pigs	3/11 (27)
Other	0/11 (0)

IQR, Interquartile range; MRSA, methicillin-resistant *Staphylococcus aureus*

### Effect on pre-emptive isolation

Median duration of pre-emptive isolation of ICU patients was 27.6, 21.4 and 96.0 hours, for patients in which isolation was discontinued upon IDI, GeneXpert and conventional culture results, respectively (Table 3). Pre-emptive isolation was discontinued upon a negative PCR result in 102 (62.6%) patients while 22 patients (13.5%) remained in isolation because of a positive (*n *= 14) or non-conclusive (*n *= 8) PCR test, 4 remained in isolation for other reasons and 35 (21.5%) patients with a negative PCR result remained in isolation during the initial phase of the IDI study conform the protocol before October 2006 (up to October 2006 results of RDT were not used to discontinue isolation measures in ICU patients, because of lack of experience with RDT). Most of the patients with positive PCR results (9/14, 64.3%) appeared MRSA negative with conventional cultures. There were no false negative PCR results.

**Table 3:** Turn-around times and duration of isolation with different screening methods; values are expressed as medians (interquartile range)

	Conventional culture^a^	BD GeneOhm™ MRSA PCR	Xpert MRSA assay^b^
	(Number = 163)	(Number = 89)	(Number = 74)
Time from start of isolation to delivery of specimen to lab, hours^c^	14.6 (0.5-19.9)	16.3 (0-19.9)^d^	5.8 (0.5-18.2)^d^
Time from arrival in the lab to definite test result, hours^e^	72.5 (67.0-96.0)	18.5 (5.3-24.0)	2.6 (1.7-20.4)
Time from start isolation to definite test result, hours	91.2 (71.0-113.8)	25.2 (18.0-43.4)	21.1 (5.3-28.3)
Isolation discontinued based on test, number^f^	48/163	40/89	62/74
Time from definite test result to discontinuation of isolation, hours^g^	0.7 (0-1.9)	1.0 (0.2-6.0)	0.2 (0-0.5)
Duration of isolation, hours^g^	96.0 (78.7-113.5)	27.6 (23.0-48.5)	21.4 (14.6-37.2)

^a^Turn-around times of conventional cultures during IDI study: time from start of isolation to delivery of specimen to laboratory: 16.3 hours (0 to 19.9); time from arrival in the laboratory to definite result: 76.1 hours (68.4 to 97.9); time from definite test result to discontinuation of isolation: 0.7 hours (0 to 1.9); time from start of isolation to definite test result: 91.9 hours (71.8 to114.0); duration of isolation 96.0 hours (86.2 to 113.5). Turn-around times of conventional cultures during GeneXpert study: time from start of isolation to delivery of specimen to laboratory: 5.8 hours (0.48 to 18.2); time from arrival in the laboratory to definite result: 69.4 hours (65.0 to 84.7); time from definite test result to discontinuation of isolation 1.0 hours (0.5 to 1.4); time from the start of isolation to definite test result 88.1 hours (70.6 to 100.1); duration of isolation: 98.9 hours (71.3 to 129.8). ^b^In 14 patients (18.9%) the exact moment of start or discontinuation of isolation was missing. ^c^When not all specimens did arrive simultaneously, time of last specimen to arrive was noted. ^d^Time for samples to arrive at the microbiology laboratories was longer during the IDI study than during the GeneXpert study, which was caused by one hospital (*n *= 35) that had an above average delivery time (median 18.0 hours) and only participated in the IDI study, and because the MRSA PCR was not performed on weekends and on holidays during the IDI study. ^e^Including weekends and public holidays when the MRSA PCR was not performed in all hospitals. ^f^In the other patients isolation measures were discontinued on discharge (*n *= 9), when the patient died (*n *= 2) or because of other reasons (*n *= 2). ^g^In patients where isolation was discontinued based on a negative test result. MRSA, methicillin-resistant *Staphylococcus aureus*; PCR, polymerase chain reaction

In 137 (84.0%) patients isolation could have been discontinued based on a negative PCR result (84.3% in IDI study and 83.8% in GeneXpert study). The estimated total number of isolation days needed based on the conventional culture strategy (and incorporating real discharge (or decease) times) was 831 days. PCR could have reduced the number of isolation days by 44.3% to 463 days, avoiding 368 isolation days.

### Cost per isolation day avoided

Costs per test were €56.22 and €69.62 for IDI and GeneXpert, respectively (Table 4). The costs per isolation day avoided were €136.04 (€5.67 per hour) in the IDI study, and €121.76 (€5.07 per hour) in the GeneXpert study. If samples in the IDI study would have been pooled resulting in < 4.6 MRSA PCRs per patient, the cost per isolation day avoided would have been lower for IDI than for the GeneXpert. As there were no false negative test results no additional costs for contact screening and isolation measures were made.

**Table 4:** Costs of adding rapid diagnostic testing of methicillin-resistant *Staphylococcus aureus *(MRSA) to the currently used MRSA policy

	Number of units	Cost/unit (€)	Additional cost (€)
IDI study			
Total cost per test^a^		56.22	
Total cost of test strategy in this study	519		29,178.18
PCR cost per patient tested (*n *= 89)		327.84	
GeneXpert study			
Total cost per test^b^		69.62	
Total cost of test strategy in this study	268		18,658.16
PCR cost per patient tested (*n *= 74)		252.14	

^a^Costs for BD GeneOhm™ MRSA PCR (assuming 200 patients per year are screened) include: assay and platform costs (price SmartCycler €41,650), depreciation (5 years) and 8% maintenance costs, personnel costs (laboratory staff and medical microbiologist), and additional costs (Liquid Stuart's swabs, gloves, consumables). A detailed description of the cost analyses has been published elsewhere [10]. ^b^Costs for Xpert MRSA PCR include: assay and platform costs (price GeneXpert €63,813.75), depreciation (5 years) and 8% maintenance costs, personnel costs (laboratory staff and medical microbiologist), and additional costs (Liquid Stuart's swabs, gloves, consumables). A detailed description of the cost analyses has been published elsewhere [10]. PCR, polymerase chain reaction.

## Discussion

In ICUs with an average prevalence of MRSA carriage among screened patients of 3.1%, the guiding of pre-emptive isolation upon RDT appeared safe and reduced the number of isolation days needed by 44% at the cost of €121.76 to €136.04 per isolation day avoided. Implementation of these techniques will markedly enhance the feasibility of control measures for nosocomial spread of MRSA. Future technical developments will probably reduce prices per isolation day avoided.

Little is known about costs of isolation measures in ICUs. In a French study performed in a medical ICU between 1993 and 1997, costs of an MRSA control policy (including contact isolation and microbiological screening) were calculated to be $655 to $705 per patient for an average length of stay of 20 days ($33 to $35 per day) [2]. Taking investment costs into account would increase costs to $1,450 per patient ($73 per day). More recent data are not available for the ICU. In other studies on nursing wards, estimated costs of an isolation day ranged from €26.34 to €46.07 [8,10,12]. From these data it is obvious that the costs per isolation day avoided as estimated in the present study are higher than the actual costs of isolation. Of note, these estimates do not include positive effects because of less logistical constraints when reducing numbers of isolation days and the costs associated with infections prevented through such an intervention.

Incremental costs are determined by costs for RDT and costs for the consequences of false negative cases. Costs for MRSA PCR are mainly influenced by the microbiological platform used and the number of PCR tests performed per patient. During the IDI study all swabs were analyzed separately while in the GeneXpert study the fourth swab and further swabs were pooled, which reduces the costs. When swabs are pooled using the IDI, this test will be less expensive than the GeneXpert. No false negative cases were observed in our study and therefore no additional costs associated with false negative screening results were included in the cost analysis. Yet, reported MRSA PCR sensitivity rates are 93.8% (95% CI 88.7 to 96.6) for IDI [13] and have ranged from 83.9% to 90.0% for GeneXpert MRSA assay [14-16]. In the Netherlands false negative results would lead to contact screenings among patients and health care workers, which would have financial consequences. The absence of false negative findings in our study, therefore, leaves some uncertainty in our calculation of extra costs attributable to PCR-based screening.

As compared to the current Dutch policy, addition of PCR-based testing for MRSA screening in ICUs could reduce the number of isolation days by 44% at a cost of €121.76 (GeneXpert study) or €136.04 (IDI study) per isolation day avoided. This is less than the reported 54% to 60% on general wards [10,17]. This lower profit for ICU patients probably is related to limitations in diagnostic capacity. ICUs patients usually have catheters, IV lines and often multiple wounds, which are all screened for MRSA according to protocol. The platforms used are not suited for the large volumes of multiple tests in a short period of time, as only 16 and 4 tests could be performed simultaneously on the Smartcycler and GeneXpert, respectively. This endorses the need for large volume testing or pooling of swabs in ICU patients to decrease unnecessary pre-emptive isolation time. Another option would be to use chromogenic agar-based screening, which has a slightly longer turn around time in the laboratory, but can be performed in large volumes and is easily implemented in routine laboratory practice, including weekend days. In general wards, chromogenic screening reduced the number of isolation days needed by 47%, which is even more than the 44% in this study, at a cost of €6.74 per isolation day avoided [10]. Although not tested in this study, chromogenic agar-based screening is also likely to be a cost-saving alternative on ICUs.

In high endemic countries, routine surveillance for MRSA carriage in ICUs, with subsequent isolation of documented carriers, has been associated with reductions in MRSA infections in ICUs and hospital-wide [18-20]. General pre-emptive isolation has been shown to reduce ICU- acquired MRSA infections in medical ICUs [6]; however, implementation is not feasible in most ICUs in high endemic areas due to a shortage of isolation rooms. As PCR-based testing decreased the number of pre-emptive isolation days by only 44%, it is unlikely that the molecular screening tests used in our study would enable implementation of pre-emptive isolation in high endemic settings. Different MRSA screening regimes, for example by varying the number of body sites tested, performing pooling of specimens or by omitting conventional cultures could minimize cost [21], and may be an appropriate alternative for high endemic countries.

The present study has several limitations, such as the quasi-experimental design of the PCR intervention study and the second-best approach that was needed to estimate the time to end of isolation measures of the 35 patients in the IDI study for whom PCR testing was not used to change isolation measures. However, exclusion of these patients did not change results.

## Conclusions

In conclusion, our study shows that PCR-based testing safely reduced the number of pre-emptive isolation days by 44% on ICUs in a low endemic setting for MRSA. Cost-effectiveness of the intervention remains to be determined. However, the benefit of PCR-based screening will increase using diagnostic procedures more suitable for large volume testing.

## Key messages

• MRSA prevalence was 3.1% (*n *= 5) in 163 patients at risk for MRSA carriage, and therefore pre-emptively isolated, in ICUs in 12 Dutch hospitals.

• Duration of pre-emptive isolation was 27.6 and 21.4 hours with IDI- and GeneXpert- based screening, respectively, and would have been 96.0 hours when based on conventional cultures.

• The number of isolation days was reduced by 44.3% with PCR-based screening at the additional costs of €327.84 (IDI) and €252.14 (GeneXpert) per patient screened.

• Costs per isolation day avoided were €136.04 (IDI) and €121.76 (GeneXpert).

• In a low endemic setting for MRSA, RDT safely reduced the number of unnecessary isolation days on ICUs.

## Abbreviations

CI: confidence interval; ICUs: intensive care units; MRSA: methicillin-resistant *Staphylococcus aureus*; NPV: negative predictive value; PCR: polymerase chain reaction; PPV: positive predictive value; RDT: rapid diagnostic testing; TATs: turn-around times.

## Competing interests

JAJW reports receiving advisory board and or consulting fees from 3M, Destiny Pharma, Novabay and Wyeth, and lecture fees from 3M and Becton Dickinson. CMJEV-G reports receiving lecture fees from Pfizer and bioMérieux. CEV reports receiving lecture fees from Pfizer and bioMérieux. AV reports advisory board fees from Cardinal Health and JohnsonDiversey, as well as lecture fees from 3M, Pfizer and bioMérieux. MJMB reports receiving advisory board fees from Ipsat therapies, 3M, Cepheid and Novartis; consulting fees from Novartis, 3M and Bayer, and lecture fees from Cepheid, Kimberly Clark and Pfizer. All other authors declare that they have no competing interests.

## Authors' contributions

MW, JK, AB, ST, AT, CVG, CV, AV, MW and MB designed the study and wrote the manuscript. MW, AW and MB performed the statistical analysis of data. MW, JK, SE, RB, AB, EE, WM, ST, AT, CVG, CV, AV, PW, MW, TZ, AW and MB participated in the collection and interpretation of data and critically revised the manuscript for important intellectual content. All authors read and approved the final manuscript.
